# Cause and Cure of Pyorrhea Alveolaris

**Published:** 1914-11-15

**Authors:** H. E. Bliler

**Affiliations:** Chicago, Ills.


### CAUSE AND CURE OF PYORRHEA ALVEOLARIS.

BY H. E. BLILER, D.D.S., CHICAGO, ILLS.

That the same bacteria of toxic matter that cause carbuncle or appendicitis and many other local morbid manifestations cause pyorrhea alveolaris, there is absolutely no question. Auto-intoxication from constipation is defined as the inadequate elimination of germ infested effete matter. In other words the retention of toxic material, poisonous gases and bacteria which permeate the entire system, attack the weakest point of resistance and cause such local morbid manifestations as pyorrhea alveolaris, carbuncle, appendicitis and many other abnormal conditions. Bacteria gravitate to points of least resistance, which has been fully verified. Clinical deductions and experiment will accord with the above facts.

The paramount importance of diet, in the prevention and treatment of disease, is daily becoming more manifest—spelling relief to many. We know by experience and clinical conclusions that stimulation and elimination are indicated, and necessary, due to our Epicurean habits. Just as long as we indulge in excesses, just so long are remedial agents essential to maintain healthy conditions, the excretory organs not being able to throw off all toxic matter.

Having personally been affected with auto-intoxication, while apparently normal, with regular elimination—my local morbid manifestations were carbuncle and pains in the appendix. After taking a thorough eliminative treatment the morbid conditions disappeared.

The acute attack of phagendenic pyorrhoea alveolaris, the real tissue destroying type, as in case No. 1, is rarely met with which clearly indicates that the same toxic gases cause pyorrhoea and other morbid phenomena, as per cases enumerated.

### WHERE THE FAULT LIES

In most all diseases a thorough treatment is indicated. With auto-intoxication from constipation it is different, in nearly all cases a single dose being administered, instead of taking an extended course of elimination, gives the patient relief, for the ailment is always present from a mild form to serious complications.

Cases which illustrate and verify my contentions.

No. 1. A young man twenty-two years of age, came to my office June 1st; complaining of soreness and bleeding of gums and a very fetid breath. Examination showed an aggravated case of phagendenic pyorrhoea with very little accumulation of sub-gingival calculus. Careful diagnosis revealed morbid phenomena, systemic disturbances being apparent. The acute systemic attack of phagendenic pyorrhoea was a vertitable fire, with a large pus pocket at angle of the jaw. I cauterized with Hcl. and H2 SO4, healing it. But the same condition would re-appear at the opposite angle, and would keep shifting back and forth like a smouldering fire. The teeth were all loose. There was no history of venereal trouble nor of any drug taken. No signs of any other disease were present. I gave him the usual local treatment with no avail. Antiseptic seemed of no benefit. After treating him for two months, I decided that I had here a case with a systemic basis. I placed him on a tonic eliminative treatment, with the result that in less than ten days all the former trouble had disappeared.

The following cases which were affected with auto-intoxication,—the cause of their morbid conditions, were all cured by the use of eliminatives.

Miss S. M. B., Age 30, with a chronic case of fetid breath; biliousness of long standing, causing severe headache; sallow complexion. No local treatment was used.

Mr. W. J. M. Age 25. Ashen color, sluggish circulation, no vigor, tri-ficial neuralgia. No local treatment used.

Mr. M. J. K. Age 21. Pimples on face of two years standing; sluggish circulation, no vigor, pale. No local treatment.

Miss S. Age 19. Very fetid breath (affected two years) teeth being kept in A1 condition. No local treatment. Referred by family physician.

Mr. S. Age 50. Fetid breath, sluggish circulation, bad condition physically. No local treatment.

Mr. R. Age 35. Had previously taken treatment four teen months. Tubercular patient—fetid breath, sluggish, ashen color, severe coughing at night. Also used with eliminatives disinfecting vapor for air passages and bronchial tubes. Progressing favorably. Lungs are clearing—cough disappearing and strength returning.

Prophylaxis should guard the oral cavity by cleansing the teeth, using efficient dentifrices and antiseptic mouth washes. Teeth aching from pulp affections are easly restored, and it is poor economy to extract them. The reflex action from pyorrhoea alveolaris and a germ laden mouth may be deleterious to the entire body. Use iodine locally, for inflamed tonsils and gums, cold sores, wounds, etc., a disinfectant of much merit.

In nasal and bronchial affections, use a disinfecting and lubricating spray by atomizing the parts with most any of the pine tar products.

The most gratifying results in the treatment of tuberculosis are obtained in this manner—combined with eliminatives.

When you cure pyorrhoea alveolaris you produce a nutritive balance, (normal health). Your recuperative powers will restore you, in proportion to your age. Life is based on elimination, and recuperation.

*Causes.* Excessive eating, negligence. Excretory organs unable to eliminate all toxic effete matter, causing systemic disturbances and imperfect metabolism.

### SYMPTOMS

Local morbid manifestations are phagendenic pyorrhoea alveolaris, sluggishness, headache, biliousness, carbuncle, pimples, languor, appendicitis tri-facial neuralgia, ashen color, and many others.

### DIET PREVENTION

Eat moderately, and of boiled or stewed meat sparingly—once daily, preferably vegetables and fruit diets—such as rice, the corn products, pineapple, apple sauce, prunes, oat meal, cereals, whole wheat and wheat bran occasionally, etc., avoiding pastries.

TREATMENT

When any morbid symptoms appear, cleanse the gastro-intestinal tract, taking internally a large dose of castor oil, followed by a vegetable cathartic, nightly for a fortnight, gradually dimininsing doses. Olive or mineral oil will assist twice weekly. Repeat above treatment quarterly and you will retain normal conditions, with good complexion and sweet breath.

---

SOME OPINIONS.

Herman Boeker, Ph. G. M. D., from personal experience and clinical observations, maintains that most all diseases are directly traceable to the Gastro-intestinal tract and that closer attention should be given to auto-intoxication as a source of disease and we will solve many of the most perplexing problems of internal medicine. Neither can we have an unalterable classification of the toxemas. Micro-organisms gravitate to those parts in which the least resistance is met. *Billroth* found bacteria in small numbers in the upper bowel segment, increasing in the intestines, and the largest number in the fecal repository, or colon. The number found in the alimentary tract is almost beyond computation in *normal* as well as abnormal conditions. *Buchard*, of Paris, lays great stress on auto-intoxication as the prime factor in most all morbid phenomena, favoring circulatory stasis of the liver, and causing, in a measure, morbid conditions and metabolic disturbances, as are found in gouty and rheumatic affections.

---

Claude L. Wheeler, A. B. M. D., professor at McGill University formerly managing editor *New York Medical Journal*, says in addition to all morbid manifestations, you will find chronic constipation (systemic stagnation) a causal factor of nervousness.

---

In the confessions of a neurasthenic, by Horace Hazeltine, author of the "City of Encounters"he says, "My appetite never was a stable feature; indigestion, fermentation and general discomfort after eating has long been one of my sorest trials."

O. Victor Limerick, M. D., New York Essayist, Medical History, says: That the first invasion of the alveolar structures (gums) is made possible by a lowering of the standard of vital resistance of the tissues, by reason of some pre-existing nutritional fault, otherwise the infection would not occur; malnutrition stands as an important predisposing cause of pyorrhea. Dentists have been too restricted in the treatment of dental disorders, generally ignoring the importance of systemic relationship.

---

Castor Oil is a soothing, healing and a painless sedative, prophylactic, refrigerant. It is unirritating to the stomach. If it is nauseating to you it is due largely to your physical (bilious) condition.

HOW IT MAY BE ADMINISTERED.

Go to the soda fountain at the drug store and ask for a glass of root beer; when it is ready to drink tell the druggist to pour in one ounce of castor oil and then drink it quickly, without stirring, and you thus avoid the unpleasant taste.

---

By taking once or twice weekly it will produce healthy conditions, and will improve materially or assist at least in the cure of most all morbid conditions. The putrefactive changes of different kinds of foods create different kinds of infection, which is thrown off by the circulation, or it attacks the weakest point of resistance, causing such ailments as carbuncle, pyorrhea alveolaris, tri-facial neuralgia, head and stomache ache, and many other things. By its use, together with liberal use of antiseptic, I cured myself of ptomaine poisoning in New York City in October, 1913. Gastritis and typhoid are of the same species of disease. The slogan of science is prevention, and life is based on nutrition and elimination. By its use you save diseased teeth, and reduce inflammation and swelling in the gums in half the time required otherwise.

Many people are under the erroneous impression that by using eliminatives, and giving constant attention, one contracts the habit. Systemic and alimentary cleanliness are as important as Oral hygiene, and is a good habit to acquire. Much credit is due to pharmaceutical companies for their furtherance of prophylaxis, by their liberal supply of literature and samples of meritorious tooth powders, pastes and disinfecting mouth lotions.

